# STRENGTHS OF GRANDPARENTING: ASSOCIATIONS WITH DAILY WELL-BEING

**DOI:** 10.1093/geroni/igz038.636

**Published:** 2019-11-08

**Authors:** Tina Vo, Helena Sidrak, Elizabeth Munoz, Chandra A Reynolds

**Affiliations:** 1 University of California Riverside, Riverside, California, United States; 2 Loma Linda University, Loma Linda, California, United States

## Abstract

Potential long-term health benefits may be afforded to grandparents in close contact with their grandchildren, although whether such benefits are visible on a day-to-day basis and among others in similar caretaking roles is unclear. We investigated how the quality and quantity of social contacts, as well as caretaker or grandparenting roles, may mediate symptom perceptions in day-to-day context in a consecutive six-day period. Older adults were recruited using an online survey service aged 59-88 years (Mage= 64.8, 55.8% grandparents, 67.8% female). Participants completed a baseline survey (N=152) followed by up to six daily surveys (N=85 of 152). Measures included daily positive and negative affect, and overall frequency of physical health symptoms. Daily social contacts were rated by participants in terms of importance/closeness of the contact. Last, participants indicated the degree of regular contact and non-custodial caretaking roles of children and their grandparent status. Findings indicated that grandparents tended to report daily contacts with closer social convoy members (B=1.40 (.437); p = .002). Moreover, a trend of reduced symptom reporting across days for grandparents was observed (B=-0.145 (.073), p=.048) adjusting for sex and age. Last, grandparents who regularly took care of their grandchildren and reported increased daily positive affect, reported fewer symptoms throughout the week (B=-0.326 (.139), p=0.02). Although modest, results indicate potentially important health benefits of grandparenting in terms of daily physical functioning that may play out over the longer term to impact health and well-being.

